# Targeting Interleukin 13 for the Treatment of Atopic Dermatitis

**DOI:** 10.3390/pharmaceutics15020568

**Published:** 2023-02-08

**Authors:** Yuliya Lytvyn, Melinda Gooderham

**Affiliations:** 1 Temerty Faculty of Medicine, University of Toronto, Toronto, ON M5S 1A1, Canada; 2 SKiN Centre for Dermatology, Peterborough, ON K9J 5K2, Canada; 3 Probity Medical Research, Waterloo, ON N2J 1C4, Canada; 4 Department of Family Medicine, Queen’s University, Kingston, ON K7L 3N6, Canada

**Keywords:** interleukin 13, lebrikizumab, tralokinumab, cendakimab, eblasakimab, atopic dermatitis

## Abstract

Atopic dermatitis (AD) is a common chronic inflammatory skin condition that has a significant impact on a patient’s quality of life and requires ongoing management. Conventional topical and systemic therapies do not target specific components of AD pathogenesis and, therefore, have limited efficacy and may be associated with long-term toxicity. Thus, AD management is challenging, with a significant proportion of patients not achieving clear skin or a reduction in pruritus. There remains a large unmet need for effective therapeutic strategies with favorable safety profiles that can be used long-term in patients with refractory AD. The emergence of targeted biological and small molecule therapies has effectively broadened available treatment options for moderate-to-severe AD. Most recently, interleukin 13 (IL-13) inhibitors were shown to be efficacious and well-tolerated, with tralokinumab already approved for use in this patient population. It is important for dermatologists to be aware of the evidence behind this emerging class of biologic agents to guide treatment choices and improve outcomes in patients with AD. The main objective of this paper is to review the current literature regarding the efficacy and safety of current and emerging anti-IL-13 monoclonal antibodies, including tralokinumab, lebrikizumab, cendakimab, and eblasakimab, for the treatment of moderate-to-severe AD.

## 1. Introduction

Atopic dermatitis (AD) is one of the most common chronic inflammatory skin conditions which impacts 15–20% of people in developed countries [1,2,3,4], with 80% of cases typically onsetting in infancy or childhood [5]. AD is characterized by localized or disseminated pruritic, xerotic, and erythematous lesions which are frequently accompanied by sleep disturbances, reduced productivity, decreased self-esteem, social isolation, depression, and suicidal ideation in severe cases [6,7,8,9,10]. Thus, the relapsing chronic symptoms of AD have a significant impact on quality of life and require ongoing long-term management [6,11,12]. Moderate-to-severe AD constitutes about 20% of the cases and requires treatment with phototherapy and/or conventional systemic immunosuppressive agents, such as corticosteroids, methotrexate, mycophenolate mofetil, cyclosporin A, and azathioprine [11,13,14,15,16]. These conventional therapies do not target specific components of AD pathogenesis and, therefore, often have limited efficacy and may be associated with long-term toxicity, posing challenges in AD management [7,8,15,17,18].

In recent years, the emergence of targeted biological and small molecule therapies has effectively broadened the available treatment options for moderate-to-severe AD. The first available biological agent for this patient population was dupilumab, an inhibitor of interleukin-4 (IL-4) receptor-α, which blocks IL-4 and IL-13 signaling and prevents the downstream inflammatory cascade [19,20,21]. Despite the demonstrated efficacy and safety of dupilumab in AD treatment, only 40% of patients were shown to achieve clear or almost clear skin in clinical trials [22,23,24], and real-world data suggest about a 70% improvement in the clinical severity score (Eczema Area and Severity Index, EASI) after 3 months of treatment [25]. Moreover, dupilumab’s use may be limited by potential associated adverse effects in 8–38% of patients, such as conjunctivitis, injection site reactions, and persistent head and neck erythema [25,26,27]. It is believed that inhibition of IL-13 is the dominant mechanism of dupilumab’s effectiveness in treating AD [28,29]. JAK-STAT pathway inhibitors were also shown to be safe and efficacious for use in AD, and, thus, abrocitinib and upadacitinib have both been approved by the European Commission, Health Canada, and the FDA for use in moderate-to-severe AD [30]. Most recently, IL-13 inhibitors have been studied for the management of AD. The main objective of this paper is to review the current literature regarding the efficacy and safety of current and emerging IL-13 monoclonal antibodies, including tralokinumab, lebrikizumab, cendakimab, and eblasakimab, for the treatment of moderate-to-severe AD.

## 2. Methods

A search was conducted from the study’s inception up until 30 October 2022, in the OVID PubMed, Medline, and Google Scholar databases and on ClinicalTrials.gov. The following specific keywords present in the title, abstract, or body were used for the search: “interleukin 13,” “lebrikizumab”, “tralokinumab”, “cendakimab”, “eblasakimab”, “atopic dermatitis,” and “eczema.” Review articles and clinical trials were included in our summary of the literature. The reference lists of included articles were reviewed for retrieval of relevant studies not identified in the original search. Only studies involving human patients published in the English language were included.

## 3. The Role of Interleukin 13 in the Pathogenesis of Atopic Dermatitis

The pathogenesis of AD is multifactorial, driven by an interplay of environmental and genetic factors that trigger inflammation, dysbiosis, and immune dysregulation of the cutaneous epidermal layer [18,31,32]. This markedly increases transdermal water loss and facilitates permeation by irritants, microbes, and allergens [32,33,34,35]. An aberrant type 2 immune response is triggered by these antigens stimulating naïve T cells to commit to the Th2 lineage [20,36,37]. This ultimately leads to the overproduction of cytokines central to atopic manifestations of AD and pruritus, namely IL-4, IL-5, IL-13, and IL-31 [38,39]. IL-4 and IL-13 contribute to epidermal barrier dysfunction by stimulating the production of immunoglobulin E (IgE), recruiting eosinophils, amplifying Th2 cell differentiation, and reducing filaggrin expression [18,37,40]. IL-4 and IL-13 also decrease anti-microbial peptide (AMP) production by keratinocytes, which predisposes the skin to *Staphylococcus aureus* colonization [20]. This may explain the altered skin microbiome in AD, whereby an abundance of *S. aureus* and a decrease in bacterial diversity further disrupt the epidermal barrier [33,41,42,43,44,45,46,47,48,49,50].

IL-4 and IL-13 are produced predominantly by activated Th2 cells, type 2 innate lymphoid cells (ILC2s), and, to a lesser extent, by type 2 CD8(+) T cells (Tc2), basophils, eosinophils, and mast cells. IL-4 and IL-13 trigger a signaling cascade via a shared heterodimeric receptor formed by IL-4 receptor α (IL-4Rα) and IL-13 receptor α1 (IL-13Rα1) [20,39,51]. The binding of either IL-4 or IL-13 to IL-13Rα1 recruits IL-4Rα, causing dimerization of the receptors, activation of Janus kinase 1 (JAK1) and tyrosine kinase 2 (TYK2), and phosphorylation of a signal transducer and activator of transcription 6 (STAT6), which promotes Th2 differentiation [8,20,52]. IL-13 also has a high affinity for the IL-13Rα2 receptor, which plays a role in the endogenous regulation of IL-13 and in the itch–scratch cycle, collagen deposition, and fibrotic tissue remodeling [34,53,54]. Thus, IL-13 may contribute to histamine-independent stimulation of afferent nerve endings and pruritus [34]. Interestingly, skin biopsy samples of patients with AD demonstrate significant overexpression of IL-13 in lesional and non-lesional skin, and only a mild IL-4 overexpression is detectable in 40% of AD lesions [28,55]. Moreover, IL-13 overexpression in peripheral blood T cells was shown to correlate with disease severity, while a decrease in its concentration correlates with improved clinical outcomes [55,56,57,58,59,60,61]. Overall, IL-13 is emerging to play an increasingly prominent role in the epidermal barrier dysfunction and inflammatory processes associated with AD [20,62].

## 4. Therapies Targeting Interleukin-13 for Management of Atopic Dermatitis

**Figure 1:**
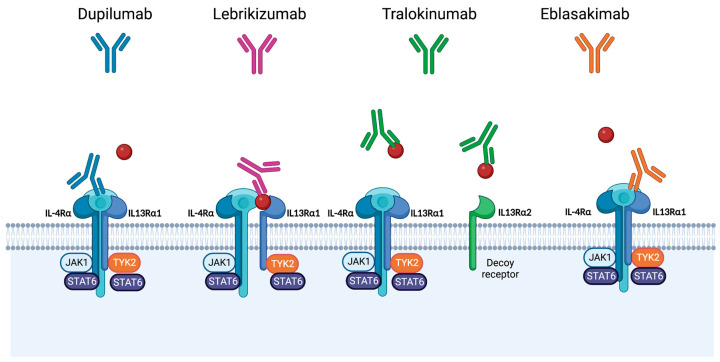
Mechanisms of IL-13 inhibition by dupilumab, lebrikizumab, tralokinumab, and eblasakimab in atopic dermatitis [18,20,63]. Dupilumab binds to the IL-4Rα subunit of IL-4 and IL-13 receptor complexes to reduce inflammatory signaling. Lebrikizumab is a fully human IgG4 that binds to IL-13 and prevents the formation of the IL-13Rα1/IL-4Rα heterodimer receptor signaling complex. Tralokinumab is fully human IgG4λ that binds to IL-13 preventing binding to the IL-13 receptor. Eblasakimab is a monoclonal antibody that targets IL13Rα1 to block IL-13 signal transduction. It, therefore, interferes with IL-4 signaling elicited via the type 2 receptor, but not the type 1 receptor. This figure was created with Biorender at www.biorender.com.

IL-13 is a promising drug target for the management of AD, with a great potential for efficacy and limited toxicity [28]. Therefore, four selective IL-13 inhibitors have emerged which are currently at various stages of development or approval. Tralokinumab has recently been approved for use in patients with moderate-to-severe AD by Health Canada, the FDA, and the European Commission. Lebrikizumab is currently in the late stages of clinical development, while cendakimab and eblasakimab are being investigated in phase II trials. See Figure 1 for the mechanisms of action of these agents.

## 5. Use of Tralokinumab to Manage Patients with Atopic Dermatitis

### 5.1. Overview of Tralokinumab

Tralokinumab is approved for use in patients 18 years of age or older with moderate-to-severe AD refractory to topical therapies. It is a fully humanized IgG4λ anti-IL-13 monoclonal antibody that competitively blocks the binding of IL-13 to both IL-13Rα1 and IL-13Rα2 receptor chains (Figure 1) [18,53,63,64,65]. In vitro studies confirmed the suppression of inflammation and restoration of the skin barrier by tralokinumab [65]. In these studies, tralokinumab treatment normalized the upregulated type 2 inflammatory markers and the downregulated genes related to terminal keratinocyte differentiation (i.e., filaggrin and loricrin) in primary human epidermal keratinocytes and human dermal fibroblasts pre-treated with IL-13 [65]. Recent investigations of the skin of patients with AD treated with tralokinumab showed increased microbial diversity, decreased Staphylococcus aureus, and increased coagulase-negative Staphylococci with treatment [66]. Therefore, tralokinumab may improve the dysbiosis of bacterial growth observed in the skin of patients with AD. The bioavailability of tralokinumab in human studies is 61%, with peak serum concentrations reached after 3–9 days (median: 5 days) and a mean half-life of 19.3 days when administered subcutaneously and 21.4 days when administered intravenously [67].

### 5.2. Clinical Efficacy of Tralokinumab in Patients with Atopic Dermatitis

There were significant clinical benefits observed with the use of tralokinumab to manage patients with AD in phase II and phase III trials (Table 1, Table 2). There have been three randomized, double-blind, placebo-controlled, 52-week, phase III trials completed with tralokinumab in patients with AD: ECZTRA 1 (NCT03131648), ECZTRA 2 (NCT03160885), and ECZTRA 3 (NCT03363854). ECZTRA 1 and 2 included 807 and 794 patients with AD, respectively. Patients were randomized to receive subcutaneous tralokinumab monotherapy with a 600 mg loading dose followed by 300 mg every other week (Q2W) or a placebo for 16 weeks [68]. The eligibility criteria included patients with AD suitable for systemic therapy with an Investigator’s Global Assessment (IGA) score of ≥3, an EASI score of ≥12, a body surface area (BSA) of ≥10%, and a pruritus numeric rating score (NRS) of ≥4 [68]. In both trials, tralokinumab showed superiority to the placebo in reaching the co-primary endpoints of achieving an IGA of 0 or 1 and an EASI of 75 by week 16 of treatment [68]. In the ECZTRA 1 trial, 15.8% of patients treated with tralokinumab achieved IGA scores of 0 or 1, compared to 7.1% of patients treated with a placebo (*p* = 0.002). EASI 75 was achieved in 25.0% of patients treated with tralokinumab compared to 12.7% of patients receiving a placebo (*p* = 0.001) [68]. Similarly, in the ECZTRA 2 trial, significantly more patients treated with tralokinumab achieved IGA 0 or 1 (22.2% vs. 10.9%, *p* < 0.001) and EASI 75 (33.2% vs. 11.4%, *p* < 0.001) compared to placebo [68]. Finally, a significantly larger proportion of patients achieved EASI 50 or EASI 90 in the tralokinumab group compared to the placebo group by week 16 [68].

Patients treated with tralokinumab who achieved primary endpoints by week 16 were re-randomized into three groups for the following 36 weeks: tralokinumab 300 mg Q2W, tralokinumab 300 mg Q4W, and placebo [68]. The patients that had a clinical response to placebos were maintained on placebos; however, they were not incorporated into the analysis after week 16 [68]. Patients that did not obtain a clinical response with placebos were treated with open-label tralokinumab 300 mg Q2W with optional topical corticosteroids [68]. If patients experienced a loss of effect between weeks 16 and 52, they were switched to the open-label tralokinumab group [68]. Rescue treatment was used to control unbearable symptoms, and patients continued in the open-label or randomized arm; however, they were considered non-responders in the final analysis [68]. By week 52, in the ECZTRA 1 trial, there were no significant differences observed in the percentage of patients reaching IGA scores of 0 or 1 without rescue medication (51% tralokinumab Q2W vs. 47% placebo, *p* = 0.68) or in the percentage of patients maintaining or reaching EASI 75 (60% tralokinumab Q2W vs. 33% placebo, *p* = 0.056) [68]. Responses in the IGA and EASI 75 in the tralokinumab group improved further at week 52 compared to week 16, suggesting peak efficacy occurs at a later time point [68]. In the ECZTRA 2 trial, IGA scores of 0 or 1 were reported in 59% of patients that continued tralokinumab Q2W treatment compared, to 25% that were re-randomized from tralokinumab Q2W to the placebo (*p* = 0.004) [68]. Similarly, an EASI 75 score was maintained in 56% of patients continuing tralokinumab Q2W treatment and 21% of patients that were switched to the placebo (*p* = 0.001) [68]. The long-term outcomes of tralokinumab treatment showed greater differences in the tralokinumab group compared to the placebo group in the ECZTRA 2 trial, which could be due to the greater use of topical corticosteroids in ECZTRA 1 by 35.8% of patients compared to 22.8% of patients in the ECZTRA 2 trial [68]. Patients treated with Q4W tralokinumab had lower frequencies of IGA 0 or 1 and EASI 75 at 52 weeks compared to the Q2W treatment schedule in both the ECZTRA 1 and ECZTRA 2 trials [68]. Greater improvements in eczema-related sleep loss and pruritus were reported in the tralokinumab group compared to the placebo group in both trials in the secondary endpoint analysis [68]. The tralokinumab group had a greater proportion of patients with pruritus NRS reductions of ≥4 (ECZTRA 1: 20.0% vs. 10.3%, *p* = 0.002; ECZTRA 2: 25.0% vs. 9.5%, *p* < 0.001), reductions in SCORAD (ECZTRA 1: −25.2 vs. 14.7, *p* < 0.001; ECZTRA 2: −28.1 vs. −14.0, *p* < 0.001), and reductions in the Dermatology Life Quality Index (DLQI) (ECZTRA 1: −7.1 vs. −5.0, *p* = 0.002; ECZTRA 2: −8.8 vs. −4.9, *p* < 0.001) [68].

ECZTRA 3 (NCT03363854) was another randomized, double-blind, placebo-controlled, 52-week, phase III trial designed to assess the efficacy and safety of tralokinumab in combination with topical corticosteroids (TCS) in patients with moderate-to-severe AD [71]. This trial included 380 patients with AD for ≥1 year with an unsatisfactory response to topical therapies or with a history of systemic medication therapy in the past year [71]. Eligibility criteria were similar to the monotherapy studies. Patients were provided with TCS throughout the study to use as needed and were randomized into a 16-week treatment with tralokinumab 300 mg Q2W after a 600 mg loading dose or a placebo [71]. At 16 weeks, significantly more tralokinumab + TCS-treated patients achieved an IGA score of 0 or 1 (38.9% vs. 26.2%, *p* = 0.015) and an EASI 75 response (56.0% vs. 35.7%, *p* < 0.001) compared to patients receiving placebo + TCS [71]. Tralokinumab + TCS was also superior to placebo + TCS in EASI 50, EASI 90, DLQI, and pruritus NRS [71]. Patients in the tralokinumab-treated group required about 50% less cumulative TCS and rescue medications compared to the placebo group (*p* = 0.004) [71].

Similar to the ECZTRA 1 and 2 trials, if patients obtained a clinical response of IGA 0 or 1 or EASI 75 at week 16 (responders) in ECZTRA 3, they were re-randomized to either continue tralokinumab 300 mg Q2W or receive tralokinumab 300 mg Q4W for another 16 weeks [71]. The remaining patients who did not achieve a clinical response (non-responders) in the placebo or tralokinumab groups initiated tralokinumab Q2W [71]. All patients continued to receive TCS as needed throughout the trial [71]. By week 32, 89.6% of patients treated with tralokinumab Q2W maintained IGA scores of 0 or 1, and 92.5% maintained the EASI 75 response without needing rescue therapy [71]. The response was maintained in the tralokinumab Q4W group in 77.6% and 90.8% of patients for IGA 0 or 1 and EASI 75, respectively [71]. There was no significant increase in TCS use in either treatment group [71]. An EASI 90 response, which was observed in approximately 60% of week 16 responders, was improved over the 32 weeks of treatment and was observed in 72.5% and 63.8% of patients treated with Q2W and Q4W tralokinumab, respectively [71]. Furthermore, a reduction of ≥4 points in the NRS pruritus score was achieved by 45.4% of the tralokinumab + TCS patients compared to 34.1% of patients in the placebo + TCS group (*p* < 0.001), as well as an improvement in the total DLQI score (−11.7 vs. −8.8, respectively, *p* < 0.001), and SCORAD scores (−37.7 vs. −26.8, respectively, *p* < 0.001) [71]. Patients who were non-responders at week 16 continued to improve with tralokinumab Q2W, with 30.5% more responders for IGA 0 or 1 and 55.8% more responders for EASI 75 at week 32 [71]. A post hoc analysis of data pooled from all patients on tralokinumab Q2W, independent from the response achieved at week 16, showed that at week 32, the EASI 50 response was maintained from week 16 to 32 in 81% of patients, while EASI 75 and EASI 90 rates progressively increased over time to 70.2% and 50.4%, respectively [72]. Finally, preliminary results of the ECZTEND trial were recently presented at the 2022 Annual Meeting of the American Academy of Dermatology. In this trial, a longer-term, 2-year continuous treatment with tralokinumab Q2W and optional TCS was studied in 1442 patients with AD [73]. A high level of response rates was sustained after 2 years of treatment, where 85.1% of treated patients achieved EASI 75, 65% achieved EASI 90, 50.5% had clear or almost clear skin measured via the IGA, 60.6% had pruritus NRS ≤ 3, and 76.4% had DLQI ≤ 5 [73].

### 5.3. Safety of Tralokinumab in Patients with Atopic Dermatitis

In the phase III trials ECZTRA 1 and ECZTRA 2, tralokinumab exhibited a similar safety profile to the phase IIb study over 16 and 52 weeks of treatment, with a similar incidence of mild to moderate adverse events in the tralokinumab and placebo groups. In ECZTRA 1, 23% of patients treated with tralokinumab reported upper respiratory tract infections (URTI; vs. 21% in the placebo group), and 7% of patients reported conjunctivitis (vs. 2% in the placebo group) [68]. Similarly, in the ECZTRA 2 trial, 10% of patients treated with tralokinumab reported URTI (vs. 9% in the placebo group) and 3% of patients reported conjunctivitis (vs. 2% in the placebo group) [68]. All cases of conjunctivitis were mild and resolved by the end of the treatment period, except for one patient who discontinued the study treatment [68]. In contrast, worsening of AD, incidents of eczema herpeticum, and skin infections were more frequently seen in the placebo group [68]. In the maintenance period, AEs occurred more frequently in the tralokinumab Q2W group than in the tralokinumab Q4W group, with a low number of events leading to permanent interruption [68]. The safety profile, including the frequency and severity of adverse events that emerged from the ECZTRA 3 trial, was comparable with that of the ECZTRA 1 and 2 trials. The tralokinumab safety profile did not appear to differ with the addition of a TCS. In ECZTRA 3, URTI was again the most common AE in 19% of patients treated with tralokinumab (vs. 11% in the placebo group), followed by conjunctivitis in 11% of tralokinumab-treated patients (vs. 3% in the placebo group) [71]. Headache was reported in 9% of tralokinumab patients compared to 5% in the placebo group [71]. Skin infections that required systemic management were more frequent in the placebo group [71]. AEs were less frequent and less severe in the tralokinumab Q4W group compared to the tralokinumab Q2W groups [71]. Four patients discontinued due to adverse events; however, none of these AEs were severe: two were due to AD worsening, one was due to herpetic eczema, and one was due to a prostate cancer diagnosis [71]. A total of 13 serious adverse events (SAEs) were recorded, with no difference between groups [71]. A recently presented analysis from ECZTRA 1, 2, 3, 5, and phase IIb trials of safety data pooled from patients with AD treated with tralokinumab Q2W with TCS for 16 weeks showed a greater proportion of patients experiencing conjunctivitis compared to placebo groups (5.4% vs. 1.9%) and a similar rate of all adverse events (65.7% vs. 67.2%, respectively), serious AEs (2.1% vs. 2.8%, respectively), mild AEs (53.2% vs. 49.0%, respectively), moderate AEs (31.5% vs. 39.0%, respectively), severe AEs (4.6% vs. 6.3%, respectively), and AEs leading to drug withdrawal (2.3% vs. 2.8%, respectively) [73]. The most frequently reported adverse events in the pooled data were viral URTIs (15.7% vs. 12.2%, respectively) and AD (15.4% vs. 26.2%, respectively) [73]. Finally, the recently presented safety analysis set from the 2-year ECZTEND trial showed 78.2% of patients reporting AEs, with 7.0% being serious AEs, 66.3% being mild, 46.3% being moderate, and 7.1% being severe [73]. Only 2.4% of patients reported AEs that led to drug withdrawal. The most frequently reported AEs were viral URTI in 20.5% of patients, AD in 17.8% of patients, and conjunctivitis in 5.3% of patients; the proportion of patients experiencing the latter was similar to previously reported trials [73]. Although tralokinumab use is associated with an increased incidence of conjunctivitis, cases reported in clinical trials with 16 weeks of treatment were mostly mild and transient [74].

## 6. Use of Lebrikizumab to Manage Patients with Atopic Dermatitis

### 6.1. Overview of Lebrikizumab

Lebrikizumab is a humanized monoclonal antibody that binds soluble IL-13 at the non-receptor binding domain with a high affinity [18]. A bound IL-13 is able to form a complex with IL-13Rα1; however, it prevents heterodimerization with IL-4Rα and prevents signal transduction [18]. Therefore, lebrikizumab inhibits the IL-4Rα–IL-13Rα1 signaling complex while continuing to regulate endogenous IL-13 via stimulation of IL-13Rα2 (Figure 1) [75]. Pharmacokinetic data for lebrikizumab are available from a large meta-analysis of 11 studies, which pooled data from 2148 patients receiving either 37.5 mg or 125 mg of lebrikizumab Q4W [76]. The bioavailability of lebrikizumab was 85.6% with a 0.156 L/day clearance, and an elimination half-life of 25.7 days [76].

### 6.2. Clinical Efficacy of Lebrikizumab in Patients with Atopic Dermatitis

Results from the first induction period of two monotherapy phase III studies for lebrikizumab management of AD have recently been presented and are consistent with those observed in the phase II trials (Table 1, Table 2) [77]. ADVOCATE 1 (NCT04146363) and ADVOCATE 2 (NCT04178967) are randomized, double-blind, placebo-controlled, parallel-group, 52-week trials that included 851 patients aged 12 years or older with moderate-to-severe AD, with inadequate responses to topical treatments, IGA scores of ≥3, and who were naïve to dupilumab and tralokinumab treatments [77]. Patients were randomized to receive a lebrikizumab 500 mg loading dose followed by 250 mg Q2W or placebo [77]. After 16 weeks of treatment in the ADVOCATE 1 trial, the co-primary endpoint IGA response of 0 or 1 was reached in 43.0% of patients in the lebrikizumab group compared to 12.8% in the placebo group (*p* < 0.001), and EASI 75 in 59.3% of patients compared to 16.4%, respectively (*p* < 0.001) [77]. Similarly, after 16 weeks of treatment in the ADVOCATE 2 trial, 33.1% of patients in the lebrikizumab group reached IGA 0 or 1 compared to 10.9% in the placebo group (*p* < 0.001), and EASI 75 was reached by 50.8% compared to 18.2%, respectively (*p* < 0.001) [77]. In both trials, a significantly greater proportion of patients achieved secondary endpoints in the lebrikizumab treatment group compared to the placebo group, such as EASI 90 (ADVOCATE 1: 38.2% vs. 9.1%, respectively, *p* < 0.001; ADVOCATE 2: 30.2% vs. 9.4%, respectively, *p* < 0.001), pruritus NRS ≥ 4 point improvement (ADVOCATE 1: 46.3% vs. 12.7%, respectively, *p* < 0.001; ADVOCATE 2: 38.3% vs. 11.3%, respectively, *p* < 0.001), sleep-loss scale score ≥ 4 point improvement (ADVOCATE 1: 38.7% vs. 5.1%, respectively, *p* < 0.001; ADVOCATE 2: 26.5% vs. 7.8%, respectively, *p* < 0.001), and DLQI ≥ 4 point improvement (ADVOCATE 1: 75.5% vs. 33.8%, respectively, *p* < 0.001; ADVOCATE 2: 64.4% vs. 34.6%, respectively, *p* < 0.001) [77].

After week 16, patients were re-randomized to either continue receiving 250 mg lebrikizumab Q2W or receive 250 mg lebrikizumab Q4W or a placebo for another 36 weeks [78]. Participants that required rescue therapy in the first 16 weeks or did not maintain an EASI ≥ 50 after 16 weeks of treatment received open-label lebrikizumab Q2W for the next 36 weeks [78]. The maintenance period (weeks 16 to 52) of the ADVOCATE 1 and ADVOCATE 2 trials has been completed and the preliminary results were presented at the European Academy of Dermatology and Venereology 2022 meeting [78]. The efficacy achieved by week 16 with the lebrikizumab treatment was reported to be maintained at 52 weeks [78]. By week 52 of treatment, clear or almost clear skin measured via IGA was achieved in a greater proportion of patients treated with Q2W and Q4W lebrikizumab regimens compared to the withdrawal group (ADVOCATE 1: 75.8% and 74.2% vs. 46.5%, respectively; ADVOCATE 2: 64.6% and 80.6% vs. 49.8%, respectively) [78]. EASI 75 was maintained in a greater proportion of patients treated with Q2W and Q4W lebrikizumab regimens compared to the withdrawal group (ADVOCATE 1: 79.2% and 79.2% vs. 61.3%, respectively; ADVOCATE 2: 77.4% and 84.7% vs. 72.0%, respectively) [78]. Overall, 81.2% and 90.3% of patients treated with lebrikizumab Q2W in ADVOCATE 1 and 2, respectively, and 80.4% and 88.1% in the Q4W group achieved a ≥4-point improvement in pruritus NRS compared to 65.4% and 67.6% of patients in the lebrikizumab withdrawal group [78].

ADhere (NCT04250337) is a randomized, double-blind, parallel-group, placebo-controlled combination trial that recruited 228 adolescents and adults aged 12 years or older with moderate-to-severe AD for 1 or more years with inadequate responses to topical or systemic treatment, an IGA score of ≥3, an EASI score ≥ 16, and a BSA ≥ 10 [79]. Patients were randomized to receive a lebrikizumab 500 mg loading dose followed by 250 mg Q2W or a placebo Q2W, where both treatment groups were treated with concomitant TCS [79]. After 16 weeks of treatment, the co-primary endpoint IGA response of 0 or 1 was reached in 41.2% of patients in the lebrikizumab + TCS group compared to 22.1% in the placebo + TCS group (*p* = 0.011), and EASI 75 was reached in 69.5% compared to 42.2% of patients, respectively (*p* < 0.001) [79]. Similarly, a significantly greater proportion of patients achieved secondary endpoints in the lebrikizumab + TCS treatment group compared to the placebo + TCS group, such as EASI 90 (41.2% vs. 21.7%, respectively, *p* = 0.008), pruritus NRS ≥ 4 point improvement (50.6% vs. 31.9%, respectively, *p* = 0.017), percent improvement in pruritus NRS (50.68% vs. 35.47%, respectively, *p* = 0.017), and DLQI ≥ 4 point improvement (77.4% vs. 58.7%, respectively, *p* < 0.036) [79].

### 6.3. Safety of Lebrikizumab in Patients with Atopic Dermatitis

Lebrikizumab is well tolerated, with a similar safety profile reported in phase II and phase III trials to date. While only preliminary long-term data are available from the phase III trials, lebrikizumab safety data appear to be similar to that of a placebo when treated for up to 16 weeks [77]. In the ADVOCATE 1 and 2 trials, the proportion of patients experiencing AEs was similar in the lebrikizumab-treated and placebo groups, with the majority of AEs being mild or moderate in severity (ADVOCATE 1: 45.4% vs. 51.5%, respectively; ADVOCATE 2: 53.0% vs. 66.2%, respectively) [77]. The proportion of patients reporting conjunctivitis in the treatment group appears to be greater compared to the placebo group in both studies (ADVOCATE 1: 7.4% vs. 2.8%, respectively; ADVOCATE 2: 7.8% vs. 2.1%, respectively) [77]. However, all conjunctivitis treatment-emergent AEs were mild-to-moderate in severity and did not lead to treatment discontinuation [77]. Infections with herpes were similar among the treatment and placebo groups (ADVOCATE 1: 3.2% vs. 4.3%, respectively; ADVOCATE 2: 2.8% vs. 4.1%, respectively) [77]. Preliminary data at 52 weeks of treatment indicated that 58.1% and 68.1% of lebrikizumab-treated patients in ADVOCATE 1 and 2, respectively, reported AEs, the majority of which were mild to moderate in severity [78]. None of the severe AEs which were reported by 3.3% and 2.6% of patients in ADVOCATE 1 and 2, respectively, were related to the study drug, as was assessed by the study investigators [78]. The most common AEs in the lebrikizumab-treated patients in ADVOCATE 1 and 2 were AD (7.8 and 10.1%, respectively), nasopharyngitis (6.8% and 9.6%, respectively), conjunctivitis (8.3% and 8.1%, respectively), herpes infections (5.0 and 4.8%, respectively), and skin infections (3.0% and 4.9%, respectively) [78].

ADhere phase III study safety data are only available up to week 28 of treatment, with 43.1% of patients in the lebrikizumab group and 34.7% of patients in the placebo group reporting AEs [79]. Conjunctivitis was reported in 4.6% of patients in the treatment arm and none in the placebo arm to date [79]. Safety data from the maintenance phases of both phase III trials will be important to improve our understanding of the safety profile of lebrikizumab longer term [79]. Nevertheless, the safety data available to date in patients with AD are consistent with those previously reported in several asthma trials of over 2000 patients since 2011 [80,81,82,83,84].

### 6.4. Future IL-13 Inhibitors

Eblasakimab and cendakimab are two more IL-13 inhibitors being developed in the pipeline and are currently being investigated in phase II trials (NCT04800315). Eblasakimab is a monoclonal antibody that targets the IL-13Rα1, a subunit of the type 2 receptor, interfering with the signaling of IL-13 and IL-4 (Figure 1). Early data presented at the Annual Meeting of the American Academy of Dermatology 2022 showed significant efficacy after 8 weeks of treatment [85]. These were preliminary reported data in a small sample size of patients with AD that were randomized to receive a placebo (n = 16) or eblasakimab at one of three doses: 200 mg (n = 4), 400 mg (n = 7), or 600 mg (n = 22) [85]. A significant reduction in EASI was reported: 50% in the 200 mg group, 63% in the 400 mg group, and 61% in the 600 mg group, as opposed to 32% in the placebo group (*p* = 0.023) [85]. EASI 50 was achieved in 50% of patients in the 200 mg group, 71% in the 400 mg group, and 77% in the 600 mg group, compared to 38% in the placebo group (*p* = 0.016) [85]. EASI 75 was achieved in 50% of patients in the 200 mg group, 57% in the 400 mg group, and 50% in the 600 mg group, as opposed to 13% in the placebo group (*p* = 0.018) [85]. Finally, the peak pruritus NRS decreased by 37% in the group of patients treated with eblasakimab 600 mg, which was a significant improvement in comparison to the 16% decrease in the group treated with placebos (*p* = 0.032) [85]. Although eblasakimab shows great promise in efficacy for the treatment of AD, this preliminary study is limited by a lack of available long-term and safety data as well as a small sample size. Thus, further trial data for eblasakimab and cendakimab are awaited.

## 7. Discussion

For many decades, conventional therapy for AD comprised topical and oral immunosuppression as well as phototherapy. Approval of dupilumab triggered a significant leap forward into an era of rapid development of targeted biological therapy for AD management. The next targeted therapies approved were the JAK inhibitors abrocitinib, baricitinib, and upadacitinib, as well as an IL-13 inhibitor, tralokinumab. It is important to carefully study each biological and small molecule class to define how the nuanced differences can be used to benefit each individual patient.

Overall, growing evidence suggests that targeted IL-13 inhibitors offer a great advantage in developing efficacious and safe management strategies for patients with moderate-to-severe AD. The two most studied IL-13 inhibitors, tralokinumab and lebrikizumab, were shown to be efficacious in phase III trials, with the response being maintained over time. Treatment with these agents also results in significant improvements in pruritus and quality of life. The detailed mechanisms of IL-13 inhibition in tralokinumab, lebrikizumab, and eblasakimab are distinct (Figure 1). Tralokinumab binds IL-13 in the IL-13Rα binding site and blocks IL-13 interaction with both IL- 13Rα1 and IL-13Rα2. Lebrikizumab binds IL-13 at the IL-4Rα- binding site and inhibits IL-4Rα and IL-13Rα1 receptors’ signaling. It is unknown whether differences in the mechanisms of IL-13 inhibition result in clinical implications, given that there are no available direct comparison studies and different study design, duration, and TCS use are present in the available phase III studies. While the mechanistic differences between tralokinumab and lebrikizumab are more subtle, dupilumab differs by targeting the IL-4Rα receptor subunit and inhibiting the binding of both IL-4 and IL13. Thus, results of current clinical programs investigating these agents will help elucidate important new insights into the roles of IL-13, IL-13Rα2, IL-13Rα1, type 1 and type 2 receptors in the regulation of inflammatory skin conditions. Moreover, direct head-to-head comparison trials are needed to elucidate the differences in the efficacy of dupilumab, selective IL-13 and JAK 1 inhibitors. While these targeted therapies offer a great advantage for an improved efficacy and safety profile, there remains a proportion of patients that do not respond to treatment, which is likely a consequence of the heterogeneity in the pathogenesis of AD. Thus, the identification of biomarkers that help predict the response to treatment is an area of unmet need. For example, in phase II tralokinumab trials, periostin and DPP-4 were used as biomarkers for IL-13 activity and were shown to be associated with a response to treatment [69]. Further development of such biomarkers will help advance the era of personalized medicine. Finally, despite all the benefits of targeted therapy that were discussed, there remain challenges with subcutaneous injection administration of biological agents for patients with needle phobia, the need for cold chain delivery, and the long half-life of these agents.

In terms of differences in safety among different biologic agents and small molecule therapies, JAK inhibitors have a class warning for thrombosis, major cardiovascular events, and malignancies that have not been reported in trials of dupilumab and selective IL-13 inhibitors. In addition, JAKs have a side effect profile that includes an increased risk of infections, such as viral infections including eczema herpeticum and herpes zoster [86]. Although head-to-head data comparing dupilumab to JAK inhibitors have been published, there are no direct head-to-head comparison trials available with the current or emerging IL-13 inhibitors. In phase III trials, all agents that inhibit IL-13, specifically dupilumab, tralokinumab, lebrikizumab, cendakimab, and eblasakimab, have been reported to have a considerably increased risk of conjunctivitis, injection site reactions, and head and neck erythema. Interestingly, there appears to be a larger risk of conjunctivitis with dupilumab (up to 22% in phase III trials) compared to tralokinumab (~11%) or lebrikizumab (~3%) [22,23,87,88]. Real-world analyses and direct head-to-head comparison trials are needed to confirm these observations. While the pathogenesis of conjunctivitis in this setting is not completely understood, it is hypothesized to be the consequence of Demodex mite proliferation, direct IL-13-mediated reduction in goblet cells, or OX40-related inflammation [87,88]. Biopsies obtained from AD patients that developed conjunctivitis while being treated with dupilumab confirmed a substantial decrease in the number of intraepithelial goblet cells [89]. Similar to dupilumab-associated conjunctivitis, this association with selective IL-13 inhibitors could be initially managed with warm compresses, artificial tears, sodium hyaluronate, or antihistamine drops while continuing the use of the biological agent. A consultation with ophthalmology is warranted if a patient develops eye pain, vision changes, purulent discharge, conjunctival scarring, or corneal involvement. Resistant or severe conjunctivitis cases may require anti-inflammatory steroid drops, calcineurin inhibitors, or cyclosporine [90].

## 8. Conclusions

Improving the knowledge of the complex inflammatory mechanisms involved in AD pathogenesis has led to an increase in the use of targeted biological therapies for the effective and safe management of this chronic skin condition. IL-13 is thought to be the main mediator implicated in the inflammation, epidermal barrier dysfunction, and pruritus associated with AD. Thus, selective IL-13 inhibitors, such as tralokinumab, lebrikizumab, and eblasakimab, have shown good efficacy in the treatment of moderate-to-severe AD. While these agents have favorable safety profiles, there remains an increased risk of conjunctivitis, requiring monitoring. Although access to biological agents remains to be a challenge for some patients, the emergence of these therapies significantly increased the effective options available to manage patients with this skin condition. Further long-term studies are ongoing to continue investigating how the use of IL-13 inhibitors can be further utilized to therapeutically manage patients with moderate-to-severe AD.

## Figures and Tables

**Table 1:** Summary of primary and select secondary outcomes for phase II clinical trials completed with tralokinumab treatment in patients with moderate-to-severe atopic dermatitis.

Clinical Trial	Eligibility (Sample Size)	Treatment Groups (Duration)	EASI	EASI 50	EASI 75	IGA 0 or 1	SCORAD 50	NRS	DLQI
***Tralokinumab***									
NCT02347176 [69]	18–75 years SCORAD ≥ 25 EASI ≥ 12 BSA ≥ 10% IGA ≥ 3 (N = 204)	45 mg tralokinumab Q2W + TCS (12 weeks)	−10.78 (*p* = 0.143)	54.3%	N/A	11.6% (*p* = 0.974)	26.9%	−1.80	N/A
		150 mg tralokinumab Q2W + TCS (12 weeks)	−15.14 (*p* = 0.027)	67.3%	N/A	19.5% (*p* = 0.281)	44.2%	−1.59	N/A
		300 mg tralokinumab Q2W + TCS (12 weeks)	−15.72 (*p* = 0.011)	73.4% (*p* = 0.03)	42.5% (*p* = 0.003)	26.7% (*p* = 0.061)	44.1%	−2.17	N/A
		Placebo + TCS (12 weeks)	−10.78	51.9%	15.5%	11.8%	19.5%	−1.03	N/A
***Lebrikizumab***									
TREBLE (NCT02340234) [70]	18–75 years EASI ≥ 14 BSA ≥ 10% IGA ≥ 3 Pruritis VAS ≥ 3 (N = 212)	Lebrikizumab 125 mg single dose	N/A	69.2%	38.5%	21.2%	N/A	N/A	N/A
		Lebrikizumab 250 mg single dose	N/A	69.5%	49.1%	28.3%	N/A	N/A	N/A
		Lebrikizumab 125 mg Q4W + TCS (12 weeks)	N/A	82.4% (*p* = 0.026)	54.9% (*p* = 0.036)	33.3%	N/A	N/A	N/A
		Placebo + TCS (12 weeks)	N/A	62.3%	34.0%	18.9%	N/A	N/A	N/A
NCT03443024 [26]	18–75 years EASI ≥ 16 BSA ≥ 10% IGA ≥ 3 (N = 280)	Lebrikizumab 250 mg loading dose + 125 mg Q4W (16 weeks)	−62.34% (*p* = 0.0165)	66.4% (*p* = 0.0554)	43.3% (*p* = 0.0610)	26.6% (*p* = 0.1917)	N/A	−35.94% (*p* = 0.0047)	N/A
		Lebrikizumab 500 mg loading dose + 250 mg lebrikizumab Q4W (16 weeks)	−69.21% (*p* = 0.0022)	77.0% (*p* = 0.0037)	56.1% (*p* = 0.0021)	33.7% (*p* = 0.0392)	N/A	−49.6% (*p* = 0.0002)	N/A
		Lebrikizumab 500 mg loading dose + 250 mg lebrikizumab Q2W (16 weeks)	−72.09% (*p* = 0.0005)	81.0% (*p* = 0.0008)	60.6% (*p* = 0.0005)	44.6% (*p* = 0.0023)	N/A	−60.63% (*p* < 0.0001)	N/A
		Placebo (16 weeks)	−41.12%	45.8%	24.3%	15.3%	N/A	4.26	N/A

EASI: Eczema Area and Severity Index; IGA: Investigator’s Global Assessment; N/A: not applicable; NRS: numerical rating scale; TCS: topical corticosteroid. *p*-values are all compared to the respective placebo group.

**Table 2:** Summary of primary and select secondary outcomes for phase III clinical trials completed with tralokinumab and lebrikizumab treatment in patients with moderate-to-severe atopic dermatitis.

Clinical Trial	Eligibility (Sample Size)	Treatment Groups (Duration)	EASI	EASI 90	EASI 75	IGA 0 or 1	SCORAD Change	NRS Change	DLQI Change
***Tralokinumab***									
ECZTRA 1 (NCT03131648)	≥18 years BSA ≥ 10% (N = 802)	Tralokinumab 300 mg Q2W (16 weeks)	−15.5 (*p* < 0.001)	14.5% (*p* < 0.001)	25.0% (*p* < 0.001)	15.8% (*p* = 0.002)	−25.2 (*p* < 0.001)	−2.6 (*p* < 0.001)	−7.1 (*p* = 0.002)
		Placebo (16 weeks)	−9.0	4.1%	12.7%	7.1%	−14.7	−1.7	−5.0
		Tralokinumab 300 mg Q2W (52 weeks)	N/A	N/A	59.6% (*p* = 0.056)	51.3% (*p* = 0.68)	N/A	N/A	N/A
		Tralokinumab 300 mg Q4W (52 weeks)	N/A	N/A	49.1% (*p* = 0.27)	38.95% (*p* = 0.50)	N/A	N/A	N/A
		Placebo (52 weeks)	N/A	N/A	33.3%	47.4%	N/A	N/A	N/A
ECZTRA 2 (NCT03160885)	≥18 years BSA ≥ 10% (N = 794)	Tralokinumab 300 mg Q2W (16 weeks)	−16.9 (*p* < 0.001)	18.3% (*p* < 0.001)	33.2% (*p* < 0.001)	22.2% (*p* < 0.001)	−28.1 (*p* < 0.001)	−2.9 (*p* < 0.001)	−8.8 (*p* < 0.001)
		Placebo (16 weeks)	−7.0	5.5%	11.4%	10.9%	−14.0	−1.6	−4.9
		Tralokinumab 300 mg Q2W (52 weeks)	N/A	N/A	55.8% (*p* < 0.001)	59.3% (*p* = 0.004)	N/A	N/A	N/A
		Tralokinumab 300 mg Q4W (52 weeks)	N/A	N/A	51.4% (*p* = 0.001)	44.9% (*p* = 0.084)	N/A	N/A	N/A
		Placebo (52 weeks)	N/A	N/A	21.4%	25.0%	N/A	N/A	N/A
ECZTRA 3 (NCT03363854)	≥18 years BSA ≥ 10% (N = 380)	Tralokinumab 600 mg loading dose + 300 mg Q2W + TCS (16 weeks)	−21.0 (*p* < 0.001)	32.9% (*p* = 0.022)	56.0% (*p* < 0.001)	38.9% (*p* = 0.015)	−37.7 (*p* < 0.001)	−4.1 (*p* < 0.001)	−11.7 (*p* < 0.001)
		Placebo + TCS (16 weeks)	−15.6	21.4%	35.7%	26.2%	−26.8	−2.9	−8.8
		Tralokinumab 600 mg loading dose + 300 mg Q2W + TCS (32 weeks)	N/A	N/A	92.5%	89.6%	N/A	N/A	N/A
		Tralokinumab 600 mg loading dose + 300 mg Q4W + TCS (32 weeks)	N/A	N/A	90.8%	77.6%	N/A	N/A	N/A
***Lebrikizumab***									
ADVOCATE 1 (NCT04146363)	≥12 years EASI ≥ 16 BSA ≥ 10% IGA ≥ 3 (N = 424)	Lebrikizumab 500 mg loading dose + 250 mg Q2W (16 weeks)	−64.75% (*p* < 0.001)	38.2% (*p* < 0.001)	59.3% (*p* < 0.001)	43.0% (*p* < 0.001)	−47.26 (*p* < 0.001)	−45.75% (*p* < 0.001)	−8.78 (*p* < 0.001)
		Placebo (16 weeks)	−26.16%	9.1%	16.4%	12.8%	−16.79	−15.24%	−2.94
		Lebrikizumab Q2W (52 weeks)	N/A	N/A	79.2% (*p* < 0.05)	75.8% (*p* < 0.001)	N/A	N/A	N/A
		Lebrikizumab Q4W (52 weeks)	N/A	N/A	77.4% (*p* < 0.05)	74.2% (*p* < 0.001)	N/A	N/A	N/A
		Lebrikizumab Withdrawal (52 weeks)	N/A	N/A	61.3%	46.5%	N/A	N/A	N/A
ADVOCATE 2 (NCT04178967)	≥12 years EASI ≥ 16 BSA ≥ 10% IGA ≥ 3 (N = 445)	Lebrikizumab 500 mg loading dose + 250 mg Q2W (16 weeks)	−60.61% (*p* < 0.001)	30.2% (*p* < 0.001)	50.8% (*p* < 0.001)	33.1% (*p* < 0.001)	−43.85% (*p* < 0.001)	−35.7% (*p* < 0.001)	−6.99% (*p* < 0.001)
		Placebo (16 weeks)	−28.22%	9.4%	18.2%	10.9%	−13.87%	−8.91%	−2.47%
		Lebrikizumab Q2W (52 weeks)	N/A	N/A	79.2% (*p* < 0.05)	64.6% (*p* < 0.001)	N/A	N/A	N/A
		Lebrikizumab Q4W (52 weeks)	N/A	N/A	84.7% (*p* < 0.05)	80.6% (*p* < 0.001)	N/A	N/A	N/A
		Lebrikizumab Withdrawal (52 weeks)	N/A	N/A	72.0%	49.8% (*p* < 0.001)	N/A	N/A	N/A
ADhere (NCT04250337)	≥12 years EASI ≥ 16 BSA ≥ 10% IGA ≥ 3 (N = 228)	Lebrikizumab 500 mg loading dose + 250 mg Q2W + TCS (16 weeks)	−76.76% (*p* < 0.001)	41.2% (*p* = 0.008)	69.5% (*p* < 0.001)	41.2% (*p* = 0.011)	−55.04% (*p* < 0.001)	−50.68% (*p* = 0.017263)	−9.79 (*p* = 0.001031)
		Placebo + TCS (16 weeks)	−53.12%	21.7%	42.2%	22.1%	−37.35%	−35.47%	−6.46

EASI: Eczema Area and Severity Index; IGA: Investigator’s Global Assessment; N/A: not applicable; NRS: numerical rating scale; TCS: topical corticosteroid. *p*-values are all compared to the respective placebo group.

## Data Availability

Not applicable.
